# MMRN1 Facilitates Renal Cell Carcinoma by Activating AMPK/MMPs Axis

**DOI:** 10.1002/cam4.71013

**Published:** 2025-06-30

**Authors:** Mingji Ye, Jian Cao, Zhihao Ming, Yu Xie

**Affiliations:** ^1^ Department of Urology, Hunan Cancer Hospital, The Affiliated Cancer Hospital of Xiangya School of Medicine Central South University Changsha China

**Keywords:** AMPK, invasion, MMRN1, renal cell carcinoma

## Abstract

**Background:**

MMRN1 is a metastasis‐associated gene that is abnormally expressed in a variety of tumors. The present study aimed to explore the role of MMRN1 in renal cell carcinoma (RCC) and its related molecular mechanisms.

**Methods:**

RNA sequencing was used to detect differential gene expression in RCC. Immunohistochemical (IHC) analysis identified the expression of MMRN1 in RCC. Statistical evaluations and the GEPIA database were conducted to examine the association between MMRN1 expression and the prognosis of RCC patients. The MTT assay was employed to assess cellular proliferative capacity, while the Transwell assay was used to evaluate metastasis. Additionally, subcutaneous tumor transplantation and lung metastasis assays in nude mice were performed to investigate the growth and metastasis of RCC cells in vivo.

**Results:**

MMRN1 was significantly upregulated in RCC. The GEPIA database revealed an association between MMRN1 and distal metastasis, as well as poor prognosis in RCC patients. The overexpression of MMRN1 was found to enhance the proliferation and metastasis of RCC cells, whereas MMRN1 knockout was opposite. Additionally, MMRN1 overexpression facilitated the growth and metastasis of transplanted tumors in nude mice models. Mechanistically, MMRN1 overexpression activated the AMPK signaling pathway in RCC, and inhibition of this pathway mitigated the observed effects.

**Conclusion:**

MMRN1 is an oncogene in RCC. MMRN1 activates MMPs through the AMPK pathway to promote the proliferation and invasion of RCC.

## 1 Introduction

Renal cell carcinoma (RCC) is a predominant form of kidney cancer arising from the epithelial cells of the kidney, constituting over 90% of renal malignancies [1, 2]. Global data from 2018 indicated approximately 4 million new cases of kidney cancer and roughly 17 million deaths attributed to the disease [1]. Presently, surgical resection remains the primary therapeutic approach, yet its efficacy is limited. Research suggests that nearly 30% of patients develop metastases following local RCC resection [3, 4, 5], with a 5‐year survival rate ranging from 60% to 70% for individuals with RCC [6]. In traditional treatment approaches, RCC exhibits low sensitivity to chemotherapy, resulting in a minimal response rate of approximately 4% to 6% [7, 8]. As a result, there is a critical need to identify key genes that regulate the recurrence or metastasis of RCC and to develop targeted pharmaceutical interventions.

MMRN1 is a member of the EMILIN/multimeric enzyme protein family and is localized within the extracellular matrix of platelets, megakaryocytes, and endothelial cells [9, 10, 11]. Platelet‐related functions of MMRN1 include platelet adhesion [12, 13] and factor V regulation [14, 15]. Deficiencies in MMRN1 have been linked to an increased risk of bleeding in platelet disorders [16]. The interaction between MMRN1 and platelets facilitates the evasion of natural killer cells by circulating cancer cells, enabling their adhesion to blood vessel walls [17, 18]. Subsequently, cancer cells are able to traverse the vascular endothelium and exit circulation, leading to the establishment of metastatic lesions [19]. The recruitment of extracellular matrix components and granulocytes via activated platelets contributes to the formation of early metastatic niches, facilitating cancer cell survival and proliferation. Additionally, activated platelets release pro‐angiogenic growth factors that play a role in regulating tumor angiogenesis [20].

In summary, this study aimed to investigate the role of MMRN1 in the proliferation and metastasis of RCC. The sequencing data revealed MMRN1 as the second most up‐regulated gene in RCC, indicating a potential role in promoting RCC development. Drawing from current literature, we propose that MMRN1 functions as an oncogene in RCC, facilitating tumor proliferation and metastasis through the AMPK pathway.

## 2 Materials and Methods

### 2.1 Clinical Specimen

In this study, a total of 106 patients who underwent nephrectomy for RCC in Hunan Cancer Hospital from January 2020 to December 2021 were selected, and cancer tissues and adjacent normal tissues (3 cm from the edge of the cancer tissue) were obtained. Inclusion criteria: the clinical and pathological diagnosis confirmed renal carcinoma; RCC patients consented to participate in the trial. Exclusion criteria: RCC patients had other serious kidney‐related diseases. Patients ranged in age from 31 to 68 years with an average age of (54.36 ± 9.3) years. None of the patients received anti‐tumor therapy before the operation, and the postoperative pathological sections confirmed RCC. This study protocol was approved by the Ethics Committee of Hunan Cancer Hospital with the informed consent of all patients and strictly in accordance with the Declaration of Helsinki.

### 2.2 Sequencing and Big Data Analysis of RCC

Three representative renal carcinoma tissues and their adjacent tissues were selected and sent to Huada Company for second‐generation sequencing. According to the sequencing data, normal samples were used as controls, and difference analysis was performed using the “limma” package in R language. The difference *p*‐values were corrected by FDR method. |log_2_FC| > 1 and adj. *p*.AL < 0.05 were used as differential gene screening criteria. In view of the differences in gene, with DAVID to analyze KEGG (https://david.ncifcrf.gov/summary.jsp). Survival analysis of differential genes in kidney cancer and normal kidney cancer samples included in TCGA and GTEx was performed using GEPIA database (http://gepia2.cancer‐pku.cn/#analysis).

### 2.3 Cell Culture

RCC cell lines (OS‐RC‐2, Caki‐1, RCC4, RCC10, and A‐498) were purchased from ATCC (USA). The cells were cultured in DMEM (Gibco, USA) containing 10% fetal bovine serum (Gibco, USA), 100 μg/mL streptomycin and 100 U/mL penicillin in a 5% CO_2_ incubator (Thermo, USA) at 37°C. When the cells were in the logarithmic stage, the cells were digested with trypsin, and the cells were inoculated into the 6‐well plate at the rate of 3 × 10^5^ cells per well. After routine culture for 24 h, the virus infection was carried out when the cell fusion degree reached about 50%. Purchase OE‐MMRN1‐wt, OE‐MMRN1‐short (secreted peptide deletion) and OE‐NC lentiviruses from Gemma to infect kidney cancer cells. Renal carcinoma cells with stable overexpression or knockout of MMRN1 were obtained after continuous screening with 2 μg/mL purinomycin (Shanghai, China) for 1 week. Ko‐MMRN1#1: CTTGGACTATACCTGAGGAT; ko‐MMRN1#2: CGTGGGAAATCGAGCCCCAC.

### 2.4 qRT‐PCR

TRIZOL kit (15596‐018, Solaibao Technology Co., LTD., China) was used to extract tissue RNA strictly according to the instructions, and RNA concentration was determined. The primers used in this study were synthesized by Shanghai Shenggong Bioengineering Co., LTD. RNA reverse transcription kit (KR116, Tiangen Biotechnology Co., LTD., Beijing, China) was stored at 42°C for 15 min, 95°C for 3 min, and 4°C. Fluorescence quantitative PCR (B21202, Bimake, Shanghai, China): 1 μL cDNA was obtained, and the reaction amplification system was 10 μL. The reaction was performed in the fluorescent quantitative PCR instrument Applied Biosystems ViiA 7 (LifeTechnologies Inc., Applied Biosystems, Foster City, CA). The amplification condition was predenatured at 94°C for 5 min, denatured at 94°C for 25 s, annealed at 60°C for 25 s, extended at 72°C for 25 s, a total of 40 cycles, terminated at 72°C for 10 min. Actin was used as the internal reference primer to calculate the relative transcription level of the target gene by using the relative quantitative method (2^−ΔΔCT^ method): ΔΔCt = ΔCt experimental group − ΔCt control group, ΔCt = Ct (target gene) − Ct (internal reference), and the relative transcription level of the mRNA of the target gene = 2^−ΔΔCt^. MMRN1 (F: GGCATTGGGCTTAACAACAGT; R: CGACATGACCCGAGTGGTT) and ACTIN (F: CATGTACGTTGCTATCCAGGC; R: CTCCTTAATGTCACGCACGAT).

### 2.5 Western Blot

Total cell or tissue protein was extracted from RIPA lysate (R0010, Solarbio, China). The protein concentration of each sample was determined by BCA kit (20201ES76, Yisheng Biotechnology Co., LTD., Shanghai). The protein of equal weight was obtained by SDS‐PAGE electrophoresis. After finishing, the protein was transferred to a PVDF membrane by the wet transfer method, and 5% skim milk powder was used at room temperature for 1 h. Primary antibodies, MMRN1 (ab314893, 1:1000, Abcam, UK), AMPK (ab32047, 1:1000, Abcam, UK), p‐AMPK (ab109402, 1:1000, Abcam, UK), MMP2 (ab97779, 1:1000, Abcam, UK), AMPK (ab109402, 1:1000, Abcam, UK), MMP9 (ab137867, 1:1000, Abcam, UK), GAPDH (ab9485, 1:1000, Abcam) were added to the membrane and was incubated overnight on a 4°C shaking table. The membrane was washed with TBST for 5 min, and diluted HRP‐labeled Goat anti‐Rabbit IgG (ab205718, 1:20,000, Abcam) was added and the set‐up was incubated at room temperature for 1 h. The membranes were fully immersed in ECL substrate solution (BMU102; Abbkibe) and then exposed using a chemiluminescence imaging system (Bio‐Rad, Hercules, USA). ImageJ software was used for protein quantitative analysis.

### 2.6 Transwell

The renal carcinoma cells were cultured in serum‐free medium for 12 h, and the cells were harvested and re‐suspended in serum‐free medium (1 × 10^5^/ml). The cells were cultured with 10% fetal bovine serum in the lower chamber, and 100 μL of cell suspension was taken and added to the transwell chamber lined with matrigel (Corning, USA). After incubating at 37°C for 24 h, the cells that did not invade the surface of the chamber were gently removed with cotton swabs, fixed with 100% methanol, and stained with 20% crystal violet (Sigma, USA). The stained invading cells were manually counted in five randomly selected areas under an inverted light microscope (CarlZeiss, Germany), and each experiment was repeated three times.

### 2.7 Immunohistochemistry

The paraffin‐embedded sections were sequentially immersed in xylene and graded ethanol solutions (100%, 90%, 80%, 75%, and 50%). Subsequently, the sections were placed in antigen retrieval buffer at 100°C for 20 min. After natural cooling, the sections were incubated with 3% hydrogen peroxide followed by 5% goat serum (C‐0005, Shanghai Haoran Biotechnology Co., LTD., Shanghai, China), each for 30 min. MMRN1 antibody (ab314893, 1:1000, Abcam, UK) was added to the tissue sections at 4°C overnight. On the next day, Polyclonal Goat anti‐Rabbit IgG (ab6785, 1: 1000, Abcam, Cambridge, UK) was added to the tissue sections at 4°C for 30 min. The sections were then incubated with DAB chromogen solution (ST033, Guangzhou Weijia Technology Co., LTD., Guangzhou, China) for 5 min, followed by PBS washing. Subsequently, hematoxylin counterstaining (PT001, purchased from Shanghai Bogu Biotechnology Co., LTD., Shanghai, China) was performed for 5 min. After thorough rinsing, the sections were dehydrated through a graded alcohol series (50%, 70%, 80%, 90%, and 100%) and xylene, and finally mounted with neutral resin. The staining results were photographed by an inverted microscope (B608; Nikon, Tokyo, Japan) and evaluated by two experienced pathologists.

### 2.8 CCK8

CCK‐8 assay kit (40203ES60, YEASEN, China) was used to detect cell proliferation. According to the density of 5000 cells/pores in each group, they were implanted in 96‐well plates with five compound pores in each group. After the cells attached to the wall, the culture was continued for 24 h. Then, 10 μL CCK‐8 reagent was added to each well and incubated at 37°C for 1 h. The absorbance value of each well at A490 nm was determined by enzyme‐labeler. Cell proliferation rate was calculated according to the following formula: proliferation rate (%) = (1 − average absorbance value of the drug treatment group)/(average absorbance value of the control group − average absorbance value of the blank hole) × 100%.

### 2.9 Tumor Mouse Models

For xenograft models, 6‐week‐old nu/nu mice were used. The mice were housed with a regular 12 h light/12 h dark cycle and were kept in an SPF environment. The animal housing environment was strictly controlled at 22°C ± 1°C and 55% ± 5% relative humidity (RH) throughout the study. 5 × 10^6^ OS‐RC‐2 cells in 100 μL PBS were inoculated into nu/nu female mice subcutaneously. Tumor growth in mice was monitored and measured every week. The tumor volumes were calculated by the equation *V* (mm^3^) = *a* × *b* ^2^/2, where “*a*” is the largest diameter and “*b*” is the perpendicular diameter. This retrospective study was approved by the ethics board of The Affiliated Cancer Hospital of Xiangya School of Medicine (Ethics No. 2022013).

### 2.10 Statistical Analysis

All data were processed using SPSS 21.0 statistical software (SPSS Inc., Chicago, IL, USA), and measurement data were expressed in the form of mean ± standard deviation. Comparison between the two groups was conducted as a *T*‐test. The chi‐square test was selected for clinical data. *p* < 0.05 indicated that the difference was statistically significant.

## 3 Results

### 3.1 MMRN1 Exhibits High Expression Levels in RCC and Is Inversely Associated With Prognosis

**FIGURE 1:**
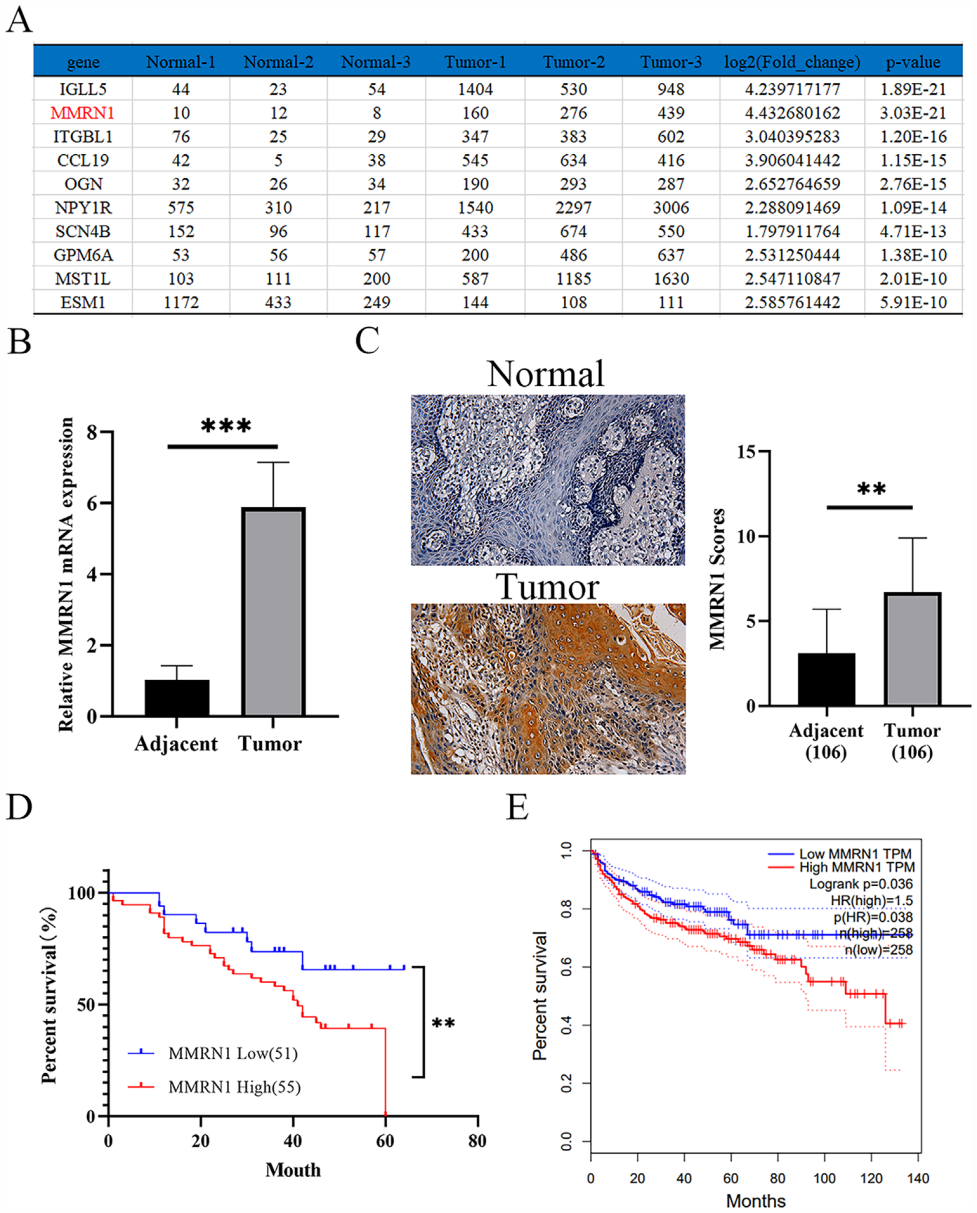
Expression of MMRN1 in RCC patients. (A) RNA sequencing found 10 genes that were most upregulated in renal cancer tissue. (B) The expression of MMRN1 in renal cancer tissues was detected by qPCR (106 cases of adjacent normal and tumor). (C) The expression of MMRN1 in renal carcinoma tissues was detected by immunohistochemistry. (D) Relationship between the expression of MMRN1 and disease‐free survival of patients. (E) Association between MMRN1 and disease‐free survival in renal carcinoma tissues in big data website GEPIA (http://gepia.cancer‐pku.cn/index.html). Compared with adjacent group or MMRN1 low group, **p < 0.01, ***p < 0.001.

Three pairs of cancerous and adjacent tissues from RCC patients were obtained and subjected to second‐generation sequencing at Huada Corporation. Analysis revealed that 10 genes, including IGLL5, MMRN1, ITGBL1, CCL19, OGN, NPY1R, SCN4B, GPM6A, and MST1L, were significantly upregulated (Figure 1A). This study highlights the limited research on MMRN1, prompting further investigation into its role in RCC. MMRN1, a platelet protein implicated in blood clotting, is also present in endothelial cells (ECs) and the extracellular matrix (ECM), potentially playing a role in cell adhesion [10, 21]. Recent research has identified MMRN1 as a differentially expressed gene (DEG) in various cancers, suggesting its potential as a cancer biomarker [22, 23, 24]. To investigate the role of MMRN1 in RCC, we obtained 106 pairs of tissue samples from RCC patients and prepared paraffin sections. The expression of MMRN1 in RCC tissues was detected by qPCR and IHC, and the results showed that the expression of MMRN1 in renal carcinoma tissues was significantly increased compared with that in adjacent tissues (Figure 1B,C). Clinical data, including age, sex, TNM stage, and survival, were collected from patients with RCC. Analysis of the expression of MMRN1 in RCC revealed a correlation with poorer prognosis in patients with high MMRN1 expression (Figure 1D). These results are consistent with data obtained from the GEPIA database (http://gepia.cancer‐pku.cn/index.html) (Figure 1E). Subsequently, the expression of MMRN1 in RCC was combined with other clinical indicators, and the results showed that the number of lymph node metastases in RCC patients with high expression of MMRN1 was higher (Table 1), and there was no significant difference with tumor size. Notably, MMRN1‐high expression was more prevalent in T3/T4‐stage RCC patients (*n* = 27), suggesting its predominant role in metastatic progression with secondary effects on cellular proliferation. Taken together, we hypothesized that MMRN1 plays a key role in the metastasis of RCC.

**TABLE 1:** MMRN1 expression associated with clinicopathology in RCC.

Clinicopathology	*n*	MRRN1 low	MMRN1 high	*p*
Ages (years)				
≤ 48	48	19	29	0.9999
> 48	58	23	35	
T stage				
I	51	31	20	0.3335
II–III	55	28	27	
N stage				
0–I	61	35	16	0.0039**
II–III	45	17	28	
TNM stage				
0–I	57	39	18	0.0184*
II–III	49	22	27	

* *p* < 0.05.

** *p* < 0.01.

### 3.2 MMRN1 Promote the Proliferation and Invasion of RCC Cells

**FIGURE 2:**
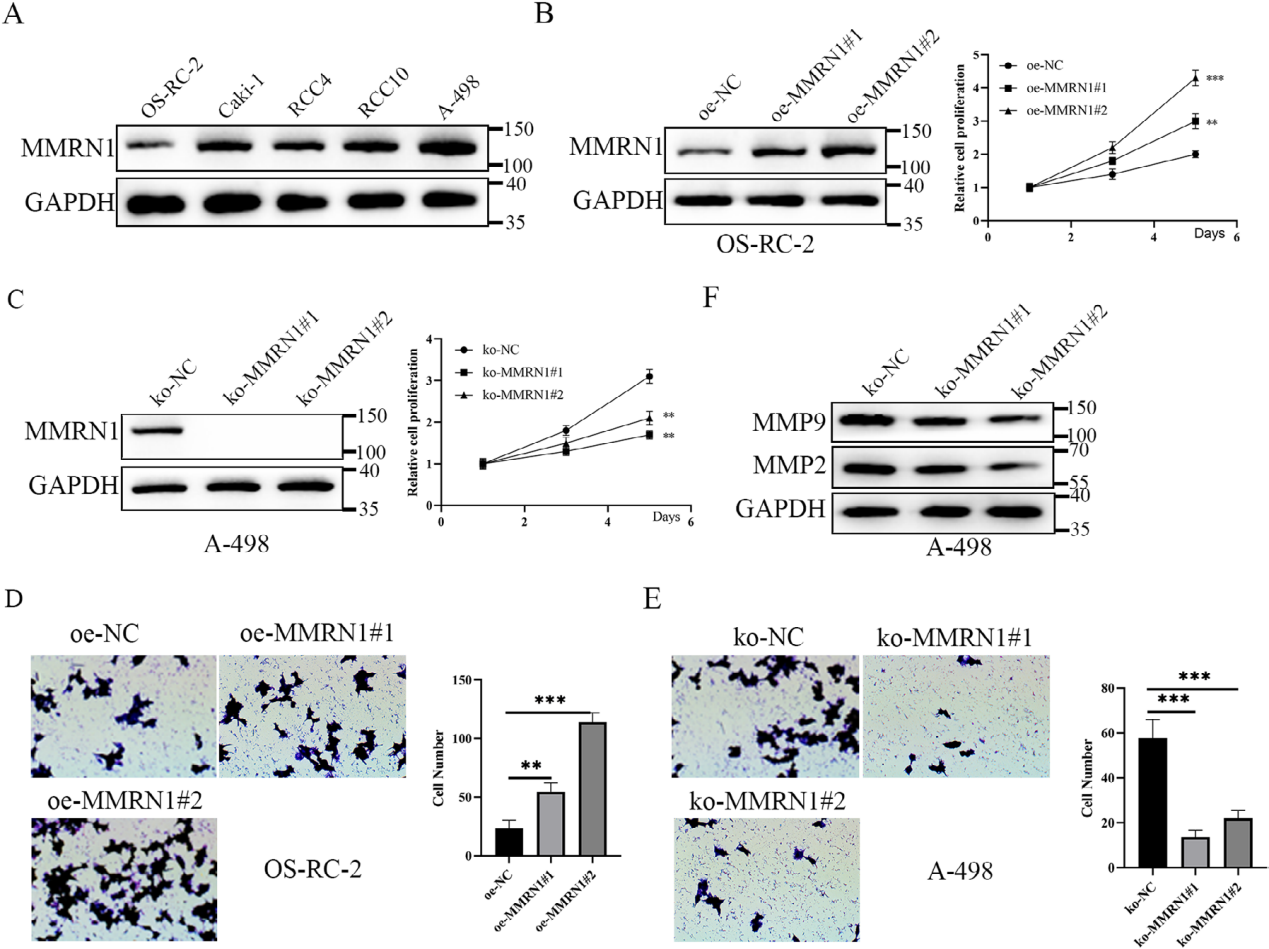
MMRN1 promotes proliferation and invasion of RCC cells. (A) The expression of MMRN1 was detected by western blot in RCC cells (OS‐RC‐2, Caki‐1, RCC4, RCC10, and A‐498). (B) CCK8 was used to detect the proliferation of OS‐RC‐2 with oversxpressed MMRN1. (C) CCK8 was used to detect the proliferation of A‐498 with MMRN1 knockout. (D) Transwell was used to detect the metastasis of OS‐RC‐2 with oversxpressed MMRN1. (E) Transwell was used to detect the invasion of A‐498 with MMRN1 knockout. (F) Western blot was used to deteced the expression of MMP2 and MMP9 in RCC cells. Compared with ko‐NC group, **p < 0.01, ***p < 0.001.

We selected five kidney cancer cells: OS‐RC‐2, Caki‐1, RCC4, RCC10, and A‐498. Western blot was used to detect MMRN1 expression in RCC cells. The result showed that MMRN1 expression was lowest in OS‐RC‐2 cells and highest in A‐498 cells (Figure 2A). Subsequent experiments were selected in these two cell lines. The oe‐NC, oe‐MMRN1#1, oe‐MMRN1#2, ko‐NC, ko‐MMRN1#1, and ko‐MMRN1#2 lentivirals were purchased and added to RCC cells. Based on the lentiviral infection efficiency, the experiments were divided into two groups: Group 1: oe‐NC, oe‐MMRN1#1, and oe‐MMRN1#2; Group 2: ko‐NC, ko‐MMRN1#1, and ko‐MMRN1#2. After screening with 5 μg/mL of purinomycin for a week, an RCC cell line with stable overexpression or knockout of MMRN1 protein was obtained (Figure 2B,C). We examined the proliferation and invasion ability of RCC cells by MTT and transwell assays. The results showed that the overexpression of MMRN1 was found to enhance the proliferation and metastasis of RCC cells (Figure 2B,D), whereas MMRN1 knockout was opposite (Figure 2C,E). Western blot results showed that MMRN1 knockout significantly suppressed the expression of MMP2 and MMP9 proteins in RCC cells (Figure 2F). These results suggest that overexpression of MMRN1 can promote the proliferation and invasion of RCC cells.

### 3.3 MMRN1 Promotes the Growth and Metastasis of RCC Cells in Nude Mice

**FIGURE 3:**
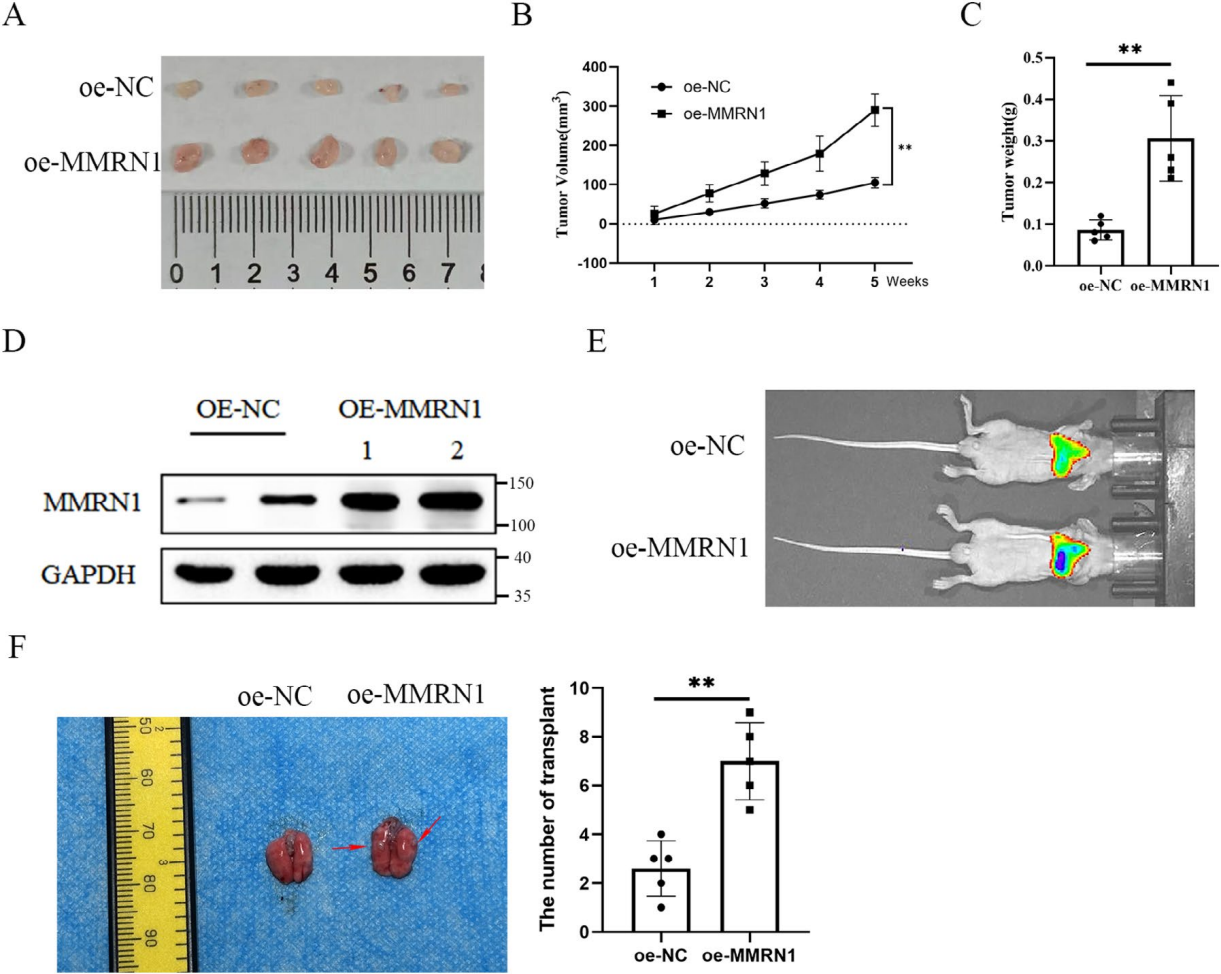
MMRN1 promotes tumor growth and metastasis in mouse models. Thirty‐five days later, mice were sacrificed and tumors were collected, photographed (A), and weighed (B). Tumor growth was monitored and measured every week (C). MMRN1 in transplanted tumors was detected by WB (D). (E) IVIS Spectrum Imager was used to detect the fluorescence in the lungs. (F) The number of nodules in the lungs was counted. Compared with the oe‐NC group, **p < 0.01.

To investigate the role of MMRN1 in RCC development, oe‐MMRN1 and oe‐NC cells were inoculated into nu/nu female mice and tumor development was monitored. Three weeks after cell inoculation, mice were sacrificed and tumors were collected (Figure 3A), weighed (Figure 3B). The tumor growth curves between control and MMRN1‐OE groups were compared and analyzed (Figure 3C). Two transplanted tumors were selected from each group; western blot results showed that MMRN1 was successfully overexpressed in transplanted tumors (Figure 3D). Next, we injected RCC into nude mice via tail vein and raised them for 28 days. After detection by IVIS Spectrum Imager, the results showed that fluorescence in the lungs of mice was significantly enhanced after overexpression of MMRN1 (Figure 3E). We collected lung tissues from mice and isolated metastatic nodules from them. The results showed that the number of nodules in the lungs was significantly increased after overexpression of MMRN1 (Figure 3F). These results suggest that MMRN1 can promote the growth of RCC cells in vivo.

### 3.4 Inhibition of AMPK Can Resist the Proliferation and Metastasis of RCC Cells Induced by MMRN1

**FIGURE 4:**
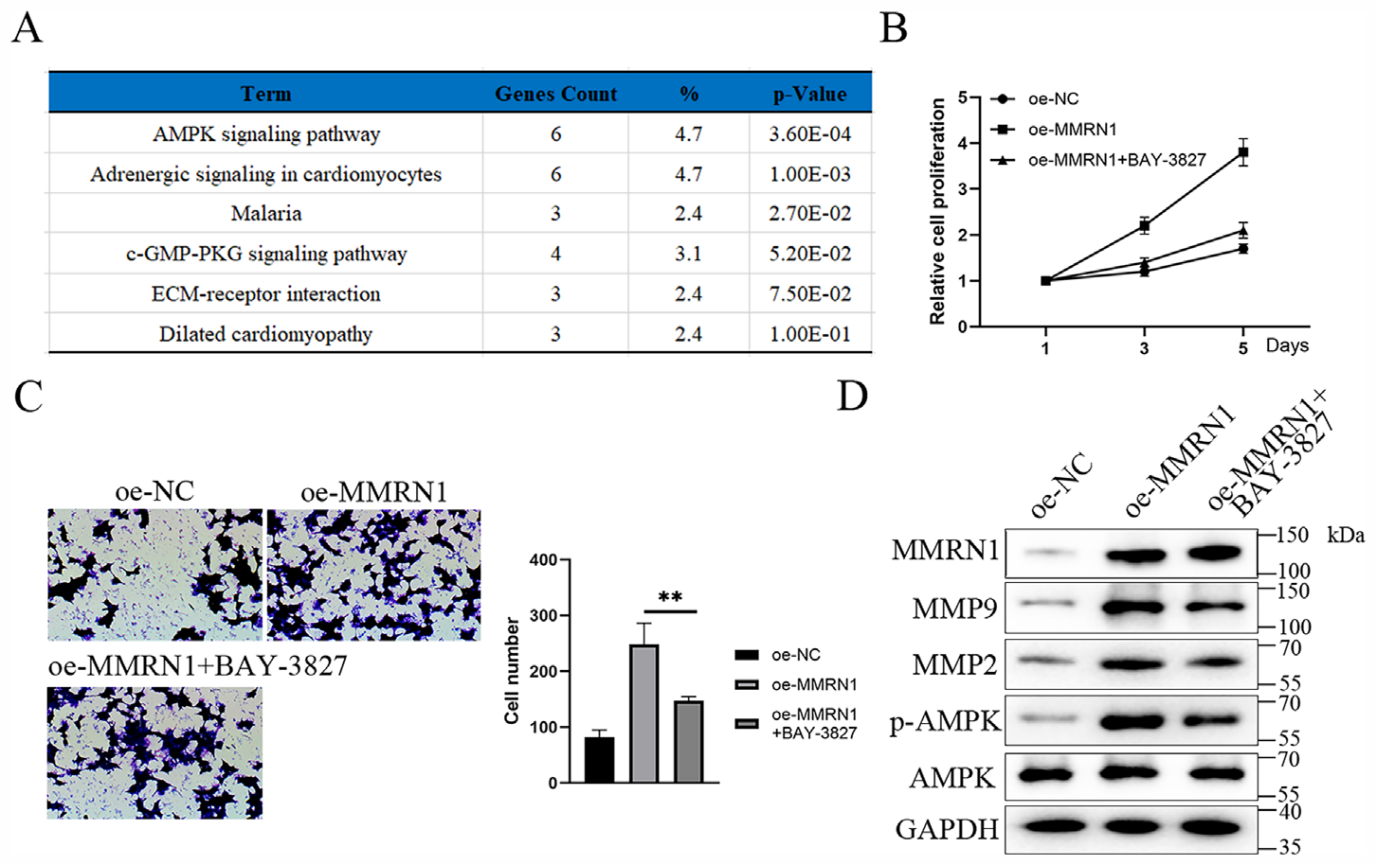
Activation of AMPK signaling pathway by MMRN1. (A) Activation of pathways in RCC. (B) CCK8 detected cell proliferation activity. (C) Transwell detected the invasion ability of cells. (D) Western blot detection of MMP2 and MMP9 expression in renal carcinoma cells. Compared with the oe‐MMRN1 group, **p < 0.01.

We analyzed the sequencing results of RCC tissues and found that the AMPK signaling pathway was significantly activated (Figure 4A). This suggests whether MMRN1 affects the AMPK signaling pathway in RCC. To determine whether MMRN1 affects the proliferation and metastasis of renal cancer cells through the AMPK signaling pathway, we performed an AMPK inhibition assay. We treated MMRN1 overexpressed cells with an AMPK inhibitor (BAY‐3827). The results of CCK8 showed that compared with the overexpressed MMRN1 group, the cell proliferation ability (Figure 4B) and migration ability (Figure 4C) were decreased in the oe‐MMRN1+ BAY‐3827 group. The result of western blot showed that overexpression of MMRN1 promoted the expression of p‐AMPK, MMP2, and MMP9 proteins. Compared with the oe‐MMRN1 group, the oe‐MMRN1+BAY‐3827 group showed significantly reduced expression of p‐AMPK, MMP2, and MMP9 proteins (Figure 4D). These results suggest that MMRN1 promotes RCC cell proliferation and metastasis through AMPK.

## 4 Discussion

RCC is a malignant tumor characterized by frequent recurrence and high mortality rates, primarily arising from renal epithelial cells [1, 4]. Despite the availability of diverse treatment modalities, including radiotherapy, chemotherapy, immunotherapy, and targeted therapy, the incidence of RCC continues to increase. Furthermore, patient prognosis remains suboptimal, primarily due to treatment resistance and associated adverse effects [7]. Consequently, there is a critical need to identify key genes responsible for the recurrence or metastasis of RCC and to develop targeted therapeutics.

In this study, we utilized clinical cases to investigate the pathogenesis of RCC by RNA sequencing on adjacent normal and tumor tissues obtained from three RCC patients. The gene differential expression analysis revealed that 10 genes, named as IGLL5, MMRN1, ITGBL1, CCL19, OGN, NPY1R, SCN4B, GPM6A, and MST1L, were significantly upregulated. MMRN1, a platelet protein known for its role in blood clotting, had been understudied in the context of cancer according to the literature. MMRN1 has been identified in endothelial cells (ECs) and extracellular matrix (ECM), potentially playing a role in cell adhesion [14, 25]. Its interaction with platelets has been shown to facilitate the escape of circulating cancer cells from natural killer cells and their adherence to blood vessel walls, ultimately leading to metastasis through extravasation [25]. Recent research has highlighted MMRN1 as a differentially expressed gene (DEG) in various cancers, suggesting its potential utility as a cancer biomarker [26, 27]. MMRN1, as an extracellular protein, has great advantages in drug‐targeted therapy. Drugs targeting MMRN1 do not need to enter the cell to work. Subsequently, we intend to find specific inhibitors for MMRN1 through a small molecule library. Efficient and accurate protocols can be achieved by combining MMRN1 inhibitors with tools that target the cell membrane, such as GalNAc [28].

The RNA sequencing data from RCC samples showed that the AMPK signaling pathway exhibits heightened activity. The AMPK pathway plays a crucial role in various cellular processes, including cell proliferation, mitochondrial biosynthesis, autophagy, ferroptosis, REDOX regulation, and energy metabolism. Previous studies have indicated that RCC can stimulate cell growth by activating AMPK, while the normal function of AMPK typically suppresses the proliferation of RCC cells [29, 30]. Studies have indicated that RCC can enhance cell growth by activating AMPK. AMPK activation typically suppresses the proliferation of RCC cells [31]. In investigations of RCC, the decrease in mitochondrial calcium uniporter (MCU) expression results in decreased ATP synthesis and AMPK activation, leading to decreased mitochondrial calcium uptake and degradation of p53, ultimately promoting cell proliferation [32].

AMPK plays a crucial role in regulating the expression and activity of matrix metalloproteinases (MMPs). Studies have shown that AMPK can influence MMP expression through various signaling pathways. For example, in mouse embryonic fibroblasts, AMPK has been found to inhibit the expression of MMP‐9 by suppressing the NF‐κB pathway [33]. In cancer cells, the regulatory role of AMPK is equally significant. Studies have found that AMPK modulates the transcriptional activity of MMP‐9 through selective autophagy and activation of Nrf2, thereby aiding cancer cells in surviving metabolic stress [34].

Additionally, phosphorylation of AMPK was significantly elevated in RCC cells after overexpression of MMRN1. However, the manner in which MMRN1 affects phosphorylation of AMPK proteins remains unknown. Then, we examined the expression of AMPK upstream proteins PKA and LKB1. Unfortunately, overexpressing MRRN1 did not activate them. We will further explore the specific mechanisms by which MMRN1 affects the AMPK pathway. Given that MMRN1 is primarily a secreted extracellular protein, we hypothesize that an unidentified membrane protein may mediate signaling between MMRN1 and AMPK. Therefore, our subsequent research will focus on two approaches: (1) Identify membrane proteins that interact with MMRN1 through co‐immunoprecipitation (co‐IP) assays and evaluate their effects on AMPK phosphorylation/activity. (2) Screen literature‐curated membrane proteins known to regulate AMPK activation, followed by validation of their functional relationship with MMRN1.

## 5 Conclusion

In summary, our study identifies MMRN1 as a significant oncogene in RCC, facilitating the degradation of MMP2 and MMP9 via activation of the AMPK signaling pathway. This process ultimately impacts the proliferation and metastasis of RCC cells. Given its role in the extracellular matrix and its correlation with metastasis, MMRN1 presents a potential target for the development of therapeutic agents for RCC.

## Author Contributions

**Mingji Ye:** data curation (lead), formal analysis (lead), investigation (equal), methodology (equal), validation (equal), writing – original draft (equal). **Jian Cao:** software (equal), validation (equal), visualization (equal). **Zhihao Ming:** resources (equal), software (equal), supervision (equal), validation (equal), visualization (equal). **Yu Xie:** conceptualization (lead), funding acquisition (lead), project administration (lead), writing – review and editing (lead).

## Conflicts of Interest

The authors declare no conflicts of interest.

## Data Availability

Data that has been analyzed and published in this research are available upon request to the corresponding author.
